# The influence of the anion exchange membrane on mass-transport limiting phenomena in bipolar interface fuel cells with Fe–N/C based cathode catalyst layers

**DOI:** 10.1039/d1ra05010a

**Published:** 2021-09-23

**Authors:** Dominik Seeberger, Pascal Hauenstein, Adrian Hartert, Simon Thiele

**Affiliations:** Forschungszentrum Jülich GmbH, Helmholtz-Institute Erlangen-Nürnberg for Renewable Energy (IEK-11) Egerlandstr. 3 91058 Erlangen Germany si.thiele@fz-juelich.de; Department of Chemical and Biological Engineering, Friedrich-Alexander University Erlangen-Nürnberg Egerlandstr. 3 91058 Erlangen Germany

## Abstract

Water management is a very important issue in low temperature fuel cells such as proton exchange membrane fuel cells (PEMFCs) or anion exchange membrane fuel cells. Within bipolar interface fuel cells, water management inhibits an even more critical role. The earlier work on bipolar interface fuel cells (BPIFCs), employing Fe–N/C on the cathode side for the oxygen reduction reaction (ORR) in an alkaline environment, demonstrated increased stability of the catalyst compared to the acidic environment of the conventional PEMFCs. However, for the BPIFCs, severe mass transport limitations (MTL) dramatically reduced the power output of the cell within a few hours. In the present work water transport processes are identified as the source of the observed MTL, after evaluating the performance data of BPIFCs, where the amount of directly deposited anion exchange membrane (AEM) material was varied. It can be seen that the BPIFCs with lower AEM content show an earlier onset of MTL than the cells prepared with higher AEM content. It is shown that the AEM can be used as a tool to regulate the influx rate of product water from the bipolar interface into the CCL and that flooding of the porous layers is identified as the main source of the observed MTL. This work paves the way for further development of BPIFCs using Fe–N/C at the cathode electrode, as novel cell design strategies can now focus exclusively on avoiding flooding phenomena.

## Abstract 2

The AEM layer content in a bipolar interface fuel cell enables the opportunity to regulate the influx rate of water into the porous layer.

## Graphical abstract

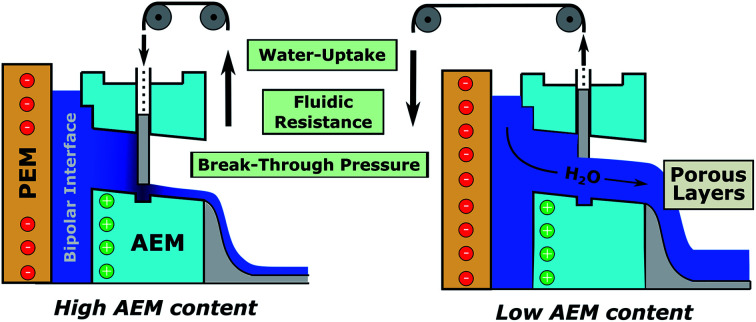

## Introduction

The hydrogen economy is a major subject of discussion on the way towards a society that is independent from fossil fuels. Within this context, hydrogen is often seen as the most promising candidate to effectively store, distribute and regain carbon-neutral, electrical energy.^1^ Polymer electrolyte fuel cells are the most commonly applied technology to convert the energy chemically stored in the form of hydrogen into electrical power.^2^ So far, platinum-group metals (PGM) are needed to catalyze the electrochemical reactions within the cells.^3^ The employment of PGMs in the cathode electrode to facilitate the oxygen reduction reaction (ORR) is one of the main cost drivers and a hindrance for the mass commercialization of the technology.^4^ Therefore, reducing the PGM content in polymer electrolyte membrane fuel cells became one of the maxims of the fuel cell community over the last decades. Currently, great expectations are placed on PGM-free ORR catalysts, with the most promising class of materials being single-atom catalysts, such as Fe–N/C.^5–8^ Although consisting of the most abundant elements being found on earth, this catalyst material can only offer significant cost reduction when meeting the requirements for catalytic activity and long-term stability.^9^ Especially meeting the latter one is still considered one of the greatest challenges for Fe–N/C based catalysts, as the acidic and corrosive nature of proton exchange membrane fuel cell (PEMFC) cathode electrodes often leads to a loss of 40–80% of the initial performance during the first 100 hours of operation.^10,11^ Replacing the acidic cathode environment by an alkaline one is often seen as a key element to mitigate the degradation of PGM-free catalyst materials.^12^ Additionally, Fe–N/C materials show intrinsically higher activities in alkaline media compared to an acidic environment.^13^

**Fig. 1:**
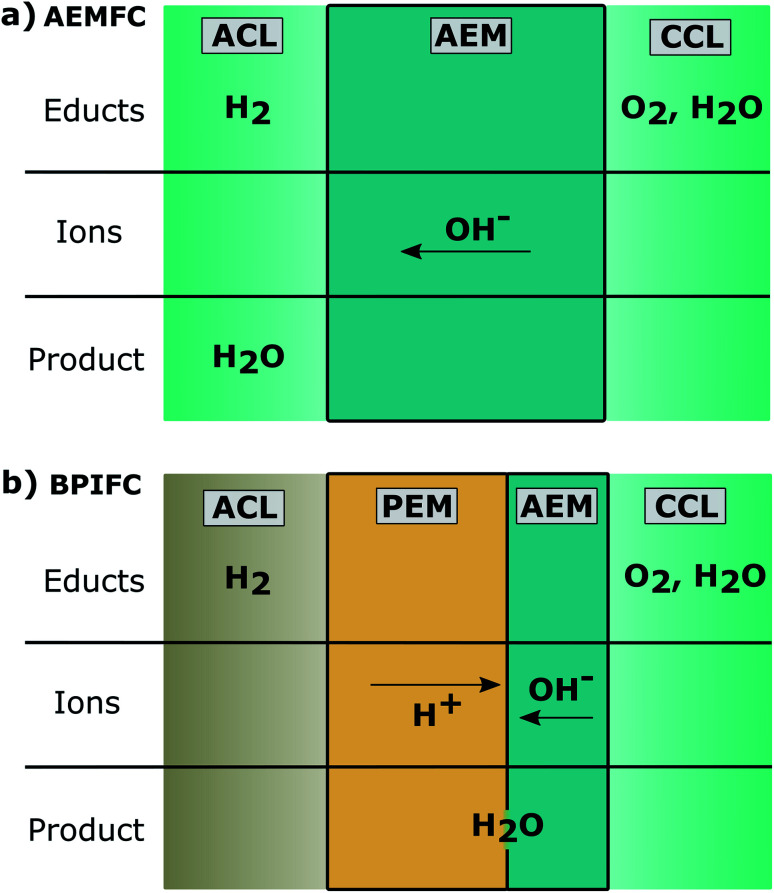
Schematic illustration of (a) an AEMFC consisting of an anode catalyst layer (ACL), an anion exchange membrane (AEM) and the cathode catalyst layer (CCL). (b) A BPIFC additionally consisting of a proton exchange membrane (PEM) and the bipolar PEM|AEM interface.

There are basically two possibilities to employ Fe–N/C as an ORR catalyst in alkaline media. In addition to the more familiar anion exchange membrane fuel cell (AEMFC), there is also the option of operating an alkaline, Fe–N/C based electrode in a bipolar fuel cell architecture. In contrast to the AEMFC, such a bipolar interface fuel cell (BPIFC) is operated at different pH values at the respective electrodes. Both fuel cell configurations and their basic operation principles are illustrated in Fig. 1.

Although the alkaline conditions in the AEMFC result in significant advantages on the cathode side, the hydrogen oxidation reaction (HOR) on the anode side is by far not as facile and efficient as in the acidic milieu of the PEMFC.^14^ In terms of numbers, it is often observed that the HOR exchange current density in alkaline medium is at least two to three orders of magnitude lower than in acidic surroundings.^15^ The anode electrode then requires significantly increased amounts of PGM-material. This often contradicts the original idea of cost and noble metal resource reduction.

As a matter of principle, BPIFC technology could compensate for some of the inherent limitations of AEMFCs: by allowing the HOR to be performed in acidic media while the ORR is maintained in an alkaline environment, in principle the BPIFC configuration can combine the ORR catalyst stability of AEMFCs and the commonly employed, low PGM content at the anode side of PEMFCs. Starting point for the considerations of this publication are our previously published results: during our previous study on the employment of Fe–N/C in BPIFCs, strong mass transport limitations (MTL) could be observed after a 15 h constant current hold in the subsequent polarization data.^16^ The phenomenon was only observed using the BPIFC configuration, whereas no signs of MTL manifested after an extended constant current hold with a conventional PEMFC configuration.^16^ It therefore seemed reasonable, that the arising mass transport resistance should originate from an exclusive feature of the BPIFC and the alkaline Fe–N/C electrode, but it was not possible to reveal its true origin(s) at that point. The mass transport limitations within the BPIFCs with Fe–N/C catalyst are most likely related to one or multiple of the following issues: one group of possible MTL sources can be associated with Fe–N/C based electrodes in general. (I) Gas-phase transport limitations due to large electrode thickness or insufficient electrode porosity (pores considered comparably dry).^17^ (II) Gas-phase transport limitations due to water accumulation in the porous network (flooding).^18^ (III) Break-down of the electrode-structure as a consequence of carbon corrosion or wrong compression.^19^ But with respect to the exclusive features of the BPIFC technology, there are additional factors to consider, that would result in strong MTL, such as. (IV) Dry-out of the CCL or the bipolar membrane during operation without external humidification.^20,21^ (V) Physical degradation of the bipolar junction, which disrupts the water recombination reaction.^22^ One unique feature of BPIFCs is that the water formation reaction does not occur at one of the electrodes, but at the junction of AEM and PEM. Therefore, the AEM|PEM interface is noticeably distinct from the other interfaces in the MEA. Previous work already demonstrated a significant, direct impact of the junction morphology on the cell's charge transfer resistance.^16^ But it is indispensable to also consider the indirect effects, *e.g.* on mass transport in the layer system the bipolar junction has on the BPIFC itself. One of the effects, induced by not having water formation in the electrodes, is a significantly altered cell water management in BPIFCs compared to conventional fuel cell systems.^23^ Balanced water management is important for all polyelectrolyte membrane fuel cell types such as anion^24^ or cation exchange membrane fuel cells.^25^ When balanced water transport management cannot be sustained, increased mass transport resistances are often the result. Intentionally manipulating the water balance in the Fe–N/C based BPIFCs on the other hand can be used as a tool to facilitate a deeper understanding of mass transport limitations. However, due to the different site of water formation, balancing the water management in BPIFCs, employing Fe–N/C based electrodes, might need different approaches than the ones considered for AEMFCs or PEMFCs. For AEMFCs the majority of challenges associated with the water management either arise from a lack of educt water at the cathode (cell dry-out) or from an excess of product water at the anode (flooding of the anode electrode).^24^ Although BPIFCs share the same cathode electrode with AEMFCs, the overall water management is changed by shifting the water formation reaction site away from the anode to the cathode-membrane interface. The closer the AEM|PEM interface to the cathode, the more likely it is that product water will be transported into the cathode electrode.^23^ Increasing water transport into the cathode also reduces the likelihood of flooding at the anode electrode, as seen in AEMFCs. For PGM-based BPIFCs, this again could allow for eliminating gas feed humidification in the BPIFC and ensuring membrane hydration and water supply for alkaline ORR from interfacial water only.^21,26^ In the case of the thick Fe–N/C based electrodes in BPIFCs, still additional gas feed humidification is required.^16^ With additional, humidified gas streams, there are now two water sources at both adjacent interfaces of the CCL.

**Fig. 2:**
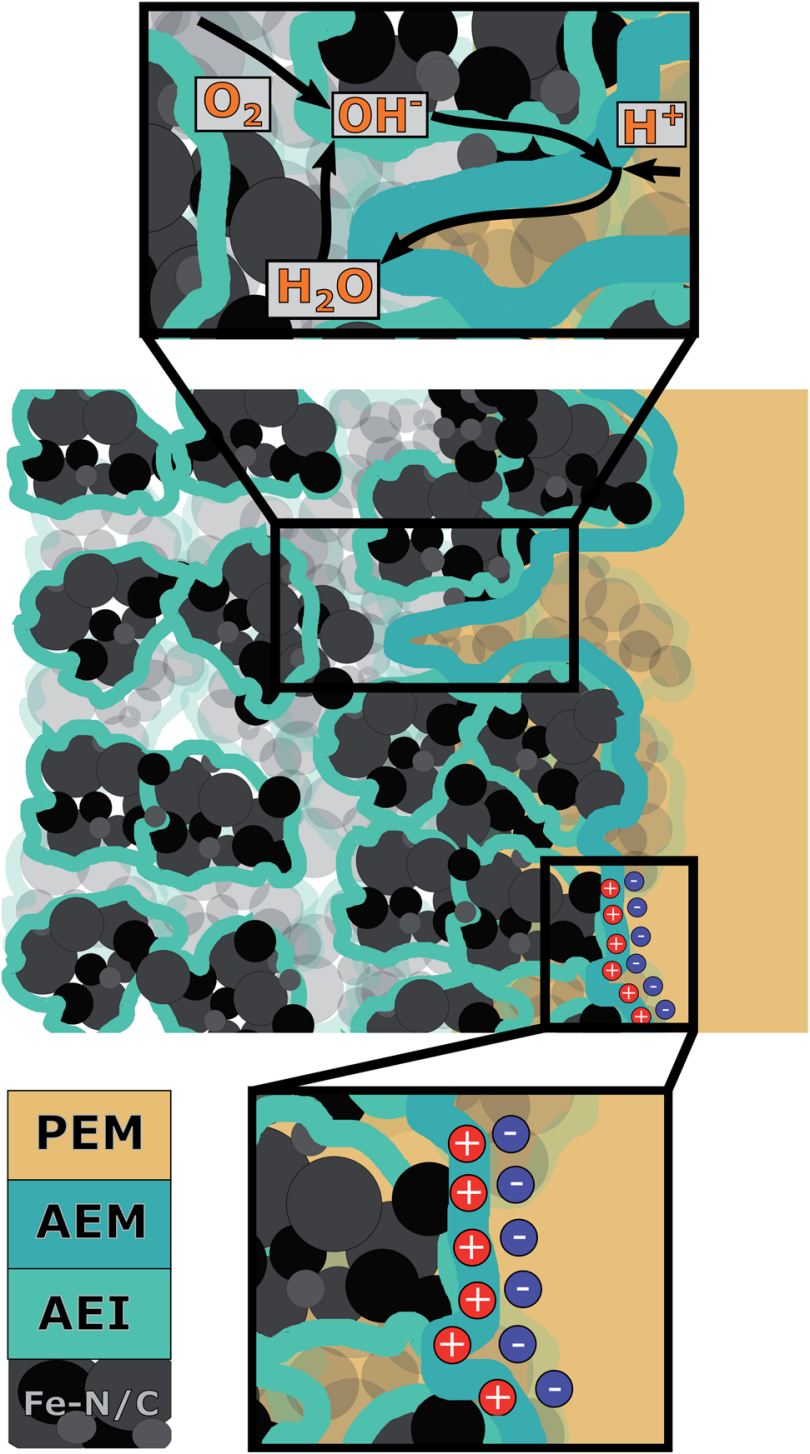
Schematic illustration of the cross-sectional view of a BPIFC, showing (from left to right) the CCL (AEI and Fe–N/C), the AEM and the PEM. Upper inset: educt- (O_2_ and H_2_O), product- (H_2_O) and ionic- (H^+^ and OH^−^) transport along the AEI, AEM and the porous media. Lower inset: illustration of the bipolar junction formed by the oppositely charged head-groups of the AEM and PEM.

To understand what caused mass transport limitations in our previous work, the interplay between the individual MEA components and their influence on cell operation behavior needs to be investigated for the case of a Fe–N/C based BPIFC. The most relevant components of such a MEA are the CCL, formed by Fe–N/C and anion exchange ionomer (AEI), the AEM and the PEM. As observed during our last study, most of the AEM dispersion infiltrates into the top of the CCL, no AEM in the conventional sense forms on the top of the CCL. The CCL pore space is still accessible after the AEM deposition, which has been shown to be one of the main advantages of using the direct membrane deposition (DMD) approach for the PEM in the next step.^16^ Depending on the deposition method including drop casting, or spray-coating of single layers^27^ or composites.^28^ For a deeper understanding of the overall system, it is therefore important to distinguish between the polymer that is incorporated into the CCL during the catalyst layer manufacturing process and the polymer that is additionally cast onto the CCL. The interaction of the two polymers within the CCL and their respective functions are shown schematically in Fig. 2.

As shown in Fig. 2, this study will distinguish between the AEI in the CCL and the AEM as the interfacial layer between the CCL and the PEM, even though both are made of identical material and no freestanding membrane is formed by the AEM layer. The AEI in the CCL is well known to affect the overall electrode morphology, like the pore size distribution.^29^ On the other hand, it provides the ionic conduction pathway towards the bipolar junction for generated OH^−^ at the catalytic active sites. The main task of the AEM is to form the bipolar interface while shielding the Fe–N/C catalyst from contact with the PEM. And as elaborated more thoroughly in previous theoretical work, the AEM layer is involved in the most important water flows of a BPIFC and therefore inhibits large potential for a better understanding of transport processes within the bipolar system.^23^ This work is focused on experimentally understanding the impact of the AEM layer on the polarization behavior of a BPIFC employing Fe–N/C electrodes and its relation to possible MTL.

### Introduction to the MEA design used in this study

**Fig. 3:**
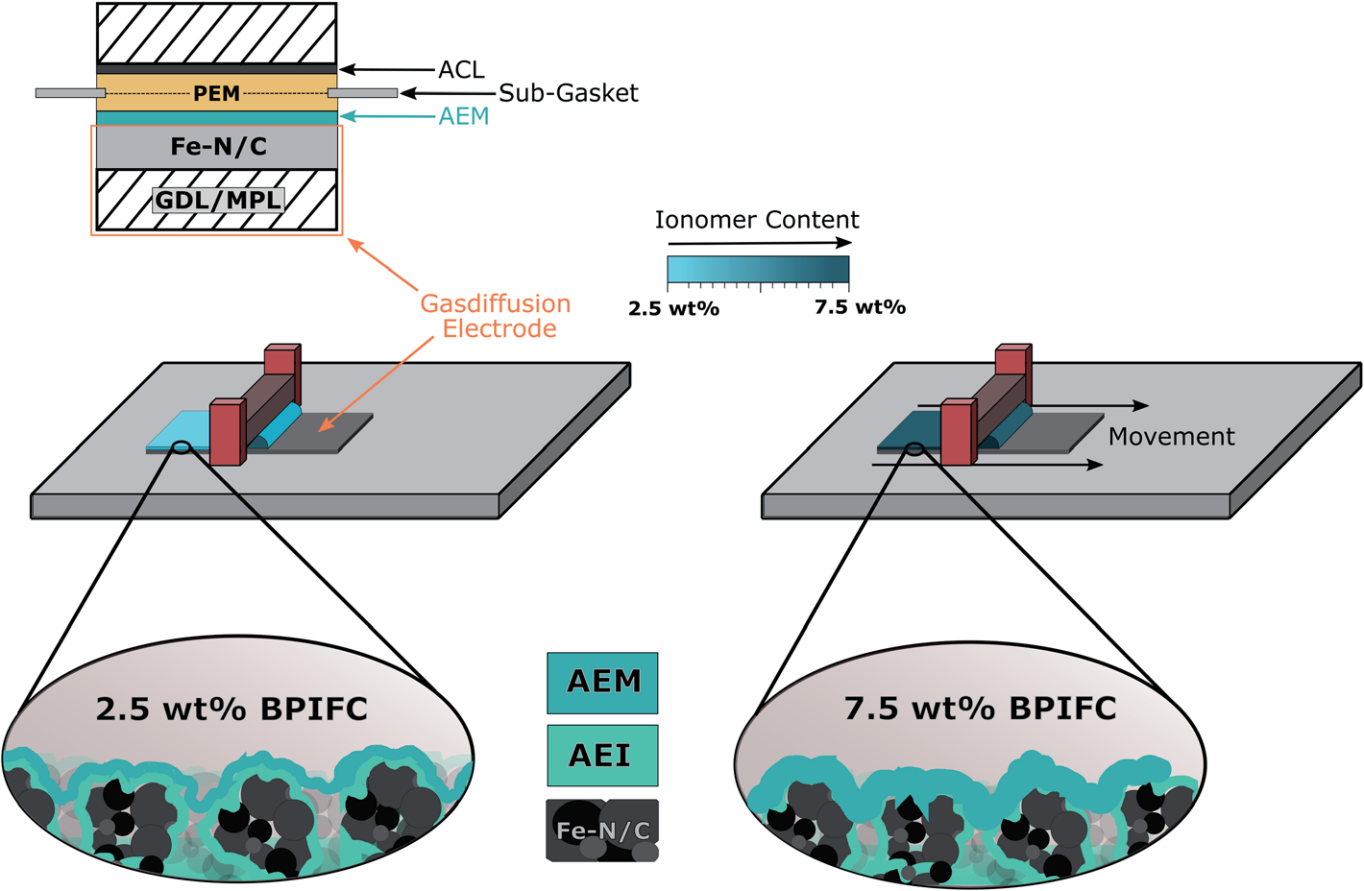
Schematic illustration of the MEA design used throughout this study and the manufacturing of the different AEM layers on the Fe–N/C based gas diffusion electrodes *via* a doctor-blading approach.

The basis for all MEAs was the deposition of the Fe–N/C based CCL onto the gas-diffusion medium to form gas diffusion electrodes (GDE) *via* doctor-blading. The GDEs were then coated with the AEI dispersion to form the AEM layer (Fig. 3). Taking our earlier work as a guide, the bipolar junction was formed by directly depositing the Nafion dispersion on the cathode half-cell to form the one half of the PEM.^16^ As commonly observed for the DMD approach, the second part of the PEM was deposited on the anode side GDE (which was kept invariant for all cells) and then assembled to a full cell as described in previous work.

**Fig. 4:**
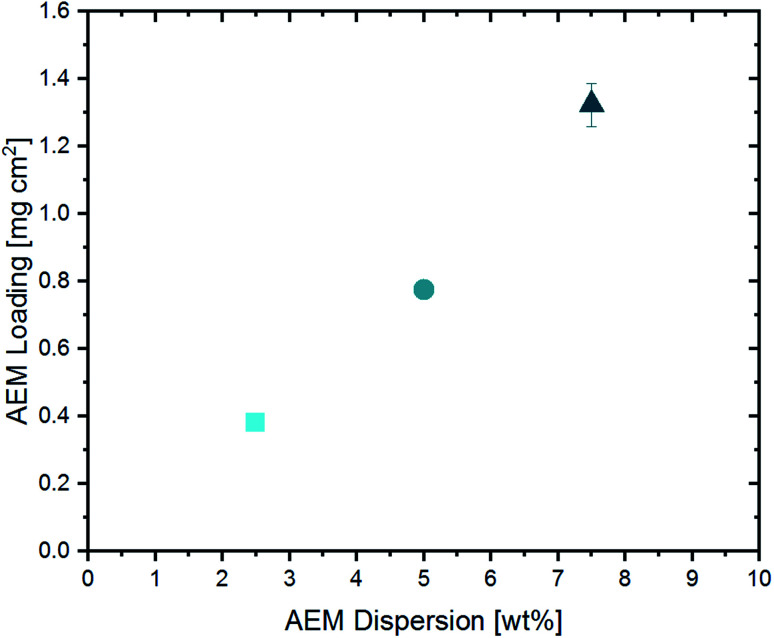
Resulting AEM loading in dependence of the solid content of the applied AEM dispersion.

The amount of ionomer forming the AEM in the upper part of the CCL was varied by changing the solid content in the AEM dispersion (2.5 wt%, 5.0 wt% and 7.5 wt%), while keeping the doctor-blade gap-height constant. The resulting AEM loadings are shown in Fig. 4.

Since the AEM content in the casting dispersion is the only difference between the three cells, they will be labeled as “2.5 wt% BPIFC”, “5.0 wt% BPIFC”, and “7.5 wt% BPIFC”.

## Results and discussion

### Starting point of the investigation: a change of polarization behavior as a function of the ionomer content in the AEM layer

**Fig. 5:**
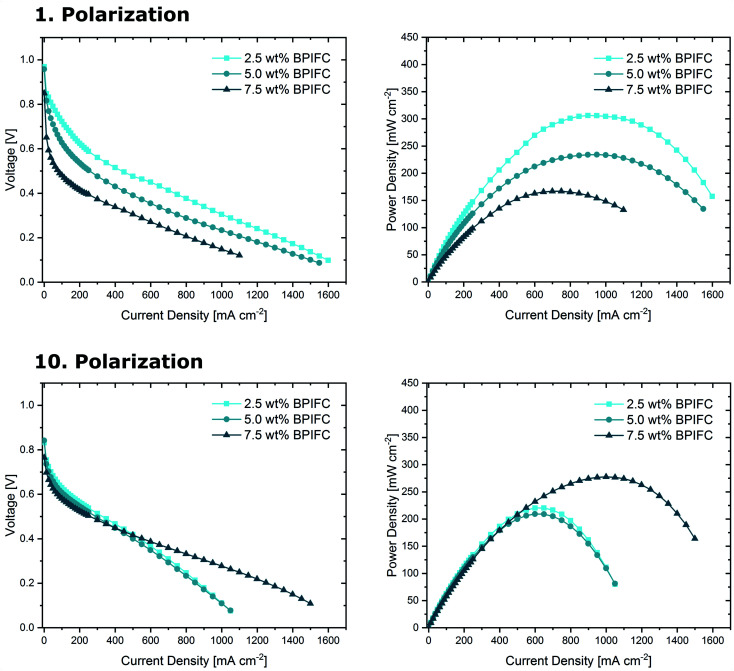
First measured polarization curves and respective power densities (top) and 10 measured polarization curves and respective power densities (bottom) for the BPIFCs with varying AEM contents. All BPIFCs were tested at 80 °C under O_2_ (0.5 L min^−1^) and H_2_ (0.5 L min^−1^), 100% RH and 200 kPa_gauge_ gas pressure.

The three MEAs were analyzed in a fuel cell setup to investigate the impact of the AEM layer on the overall device performance. Initially, the base performance of the cells was determined under pure oxygen conditions over the course of ten polarizations (Fig. 5).

**Fig. 6:**
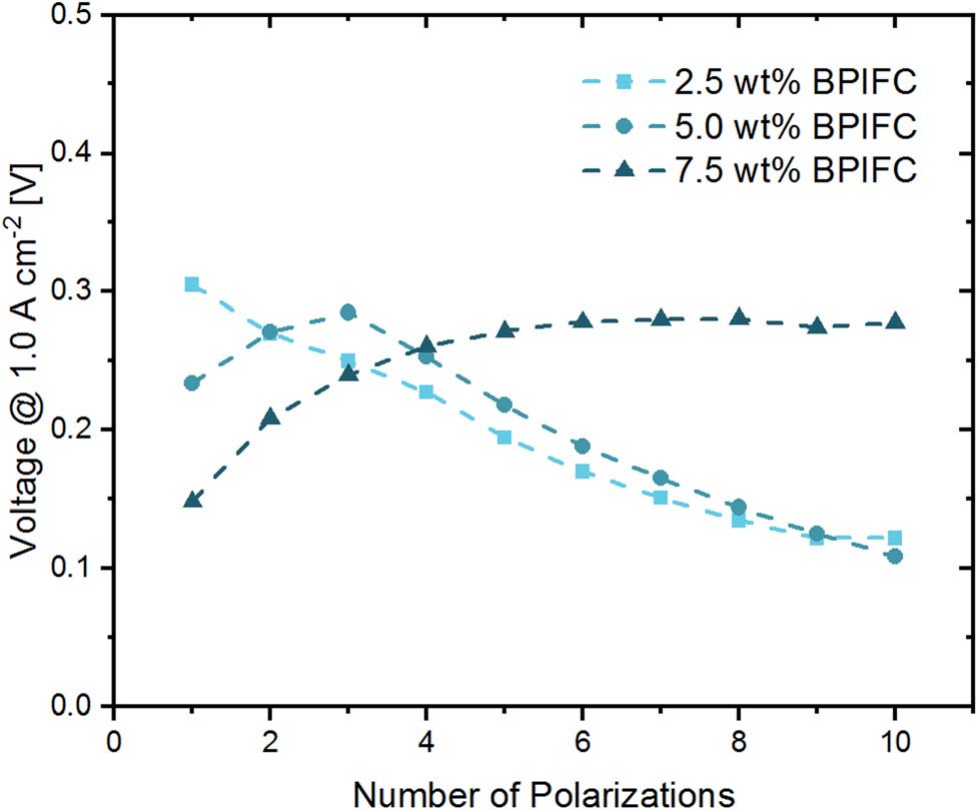
Development of the voltage at 1.0 A cm^−2^ over the course of ten polarizations.

The 2.5 wt% BPIFC showed the best initial performance (306 mW cm^−2^), followed by the 5 wt% BPIFC (234 mW cm^−2^), whereas the lowest initial performance (167 mW cm^−2^) was observed for the 7.5 wt% BPIFC. After the first polarization, none of the *I*–*V* characteristics indicated any evidence of mass transport limitations. After re-analyzing the cell performance after ten consecutive polarizations, significant changes were visible for all three MEAs. The BPIFC prepared from the 7.5 wt% AEM dispersion now showed the highest power output of the three cells (271 mW cm^−2^), which corresponds to an absolute power density improvement of more than 60% over the course of ten polarizations. By contrast, the *I*–*V* curves of the other two cells (2.5 wt% and 5 wt%) now displayed characteristics of MTL for current density values exceeding about 500 mA cm^−2^. This reduced the maximum power density of both cells to 220 mW cm^2^ and 210 mW cm^−2^ respectively. Despite the nearly identical polarization characteristics of the 2.5 wt% BPIFC and the 5.0 wt% BPIFC after ten polarizations, major differences became visible when plotting the resulting voltage at 1.0 A cm^−2^ against the number of polarizations (Fig. 6).

For the 2.5 wt% BPIFC, the voltage in the high current density region experienced significant losses after each polarization. The opposite effect was seen for the 7.5 wt% BPIFC, which displayed a voltage increase over the first five polarizations before entering a nearly steady plateau. For the 5.0 wt% BPIFC, the voltage changes were positive over the initial polarizations, but then converged to the voltage values of the 2.5 wt% BPIFC. In other words, the strong mass transport limitations seen for 2.5 wt% BPIFC and 5.0 wt% BPIFC in the final polarization curve appeared slightly delayed for the cell with medium AEM loading.

### What is the origin of the mass transport limitation characteristics?

Based on the observed phenomenon the obvious question to pose is why the polarization behavior changes as a function of the cycling number. As the cell performance increases for certain configurations, a pure catalyst layer degradation effect seems unlikely. Also a break-down of the electrode structure can most likely be excluded, as all cells shared identical CCL properties. A reduced influx of water into the CCL can also manifest as a distinct mass transport-limiting phenomenon in the polarization data. However, since mass transport limitations became more evident at the lower AEM content and were not observed at the highest AEM content while the gas supply was maintained at 100% RH in both cases, water deficiency in the CCL can also most likely be ruled out as a source of mass transport limitation. The remaining most likely options for the occurrence of the observed changes in polarization behavior are: (i) gas-phase transport limitations in the bulk electrode or (ii) the delamination of the bipolar interface and (iii) the accumulation of liquid water (flooding) within the porous layers. These three options will be thoroughly discussed in the following section, to answer the question how the AEM loading influences the polarization behavior of the BPIFCs.

#### (i) Bulk electrode gas-phase transport limitations

**Fig. 7:**
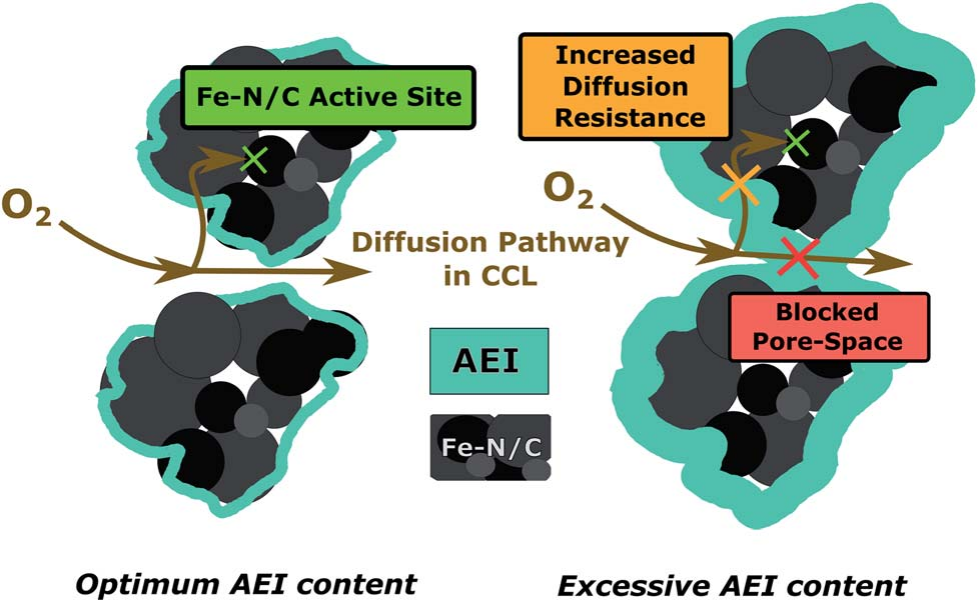
Influence of the AEI content in the CCL on the bulk electrode gas-phase transport properties.

For Fe–N/C-based electrodes, bulk electrode gas-phase transport limitations are often considered as a possible origin of mass-transport limitation phenomena. This is usually related to the large thickness or insufficient porosity and thus high fluidic resistance of the oxygen transport in the PGM-free electrode.^17,29^ For PEMFCs using the same class of Fe–N/C catalysts, it has been shown that an excessive increase in the ionomer content in the CCL can reduce the overall electrode porosity and therefore cause severe gas-phase transport limitations at the electrode due to ionomer swelling during operation.^29^ Since the AEM dispersion nearly completely infiltrates into the upper part of the CCL during the manufacturing process, it is reasonable to suspect changes in the electrode morphology, which could lead to severe mass transport limitations in the gas phase, which is schematically illustrated in Fig. 7.

**Fig. 8:**
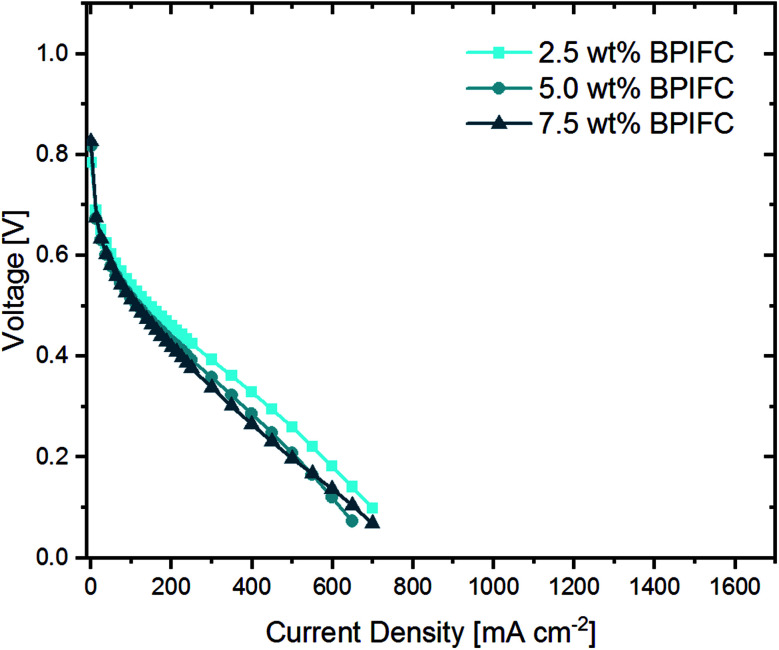
Polarization curves with synthetic air as oxidant for the BPIFCs with varying AEM contents. All BPIFCs were tested at 80 °C under air (0.75 L min^−1^) and H_2_ (0.5 L min^−1^), 100% RH and 200 kPa_gauge_ gas pressure.

From literature, it is well known that gas-phase transport limitations are enhanced when air is used as an oxidant instead of pure oxygen.^2,29^ For that reason, for the very same samples, the oxidant was switched to synthetic air after ten polarizations in oxygen and additional five polarizations were recorded and the last polarization (Fig. 8) was compared to the data collected under pure oxygen conditions.

When comparing the air polarization data with the oxygen data, the obvious cell voltage decay at higher current densities hinting towards a limited mass transport disappear. In fact, the 2.5 wt% BPIFC, which showed the strongest evidence of mass transport limitations under H_2_/O_2_-conditions, provided the highest performance under H_2_/air-conditions. However, even the 7.5 wt% BPIFC, which contained the highest amount of additionally added ionomer as AEM, showed no signs of mass transport limitations under air conditions. The performance of all three cells is comparable and no direct influence of the AEM loading was seen in the air polarization data. Two things can be deduced from these observations. First, it means that the mass transport limiting phenomena visible when oxygen is used are not induced by the bulk electrode structure itself (*e.g.* reduced porosity due to swelling of the ionomer). Otherwise, the mass transport limiting phenomena should have been enhanced if air had been used instead of pure oxygen. Second, even with the addition of up to 1.4 mg cm^−2^ AEI (7.5 wt%) as AEM material that has been almost completely infiltrated into the upper part of the CCL, the air performance is almost unchanged compared to the case where less than one-third of the ionomer amount is added. This indicates only a minor influence of the AEM layer on the gas phase transport behavior within the electrode.

#### (ii) Ionic transport limitations at the bipolar junction

**Fig. 9:**
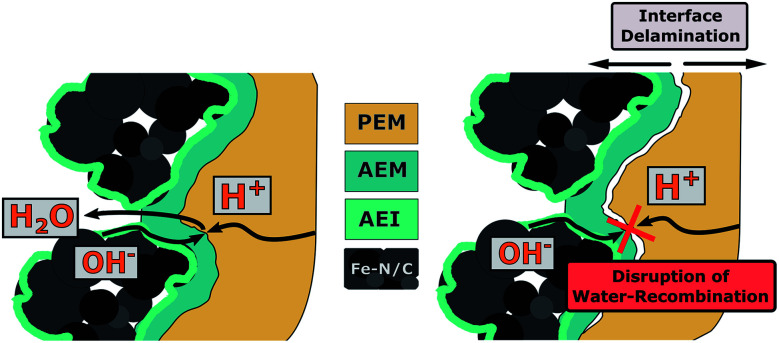
Bipolar junction degradation as possible origin of mass transport limiting phenomena.

One of the main functions of the AEM is the formation of the bipolar junction with the PEM, but also extends the ionic pathways of the CCL towards the interface. A reduction of the AEM fraction could therefore lead to deteriorated ion transport properties or to changes of the adhesion (and interface stability) between AEM and PEM at the bipolar junction. Both, the ionic transport resistance across the AEM and the interfacial contact resistance of the bipolar junction are included in the measurable value of the ohmic resistance (*R*_ohmic_).^2^ For PEMFCs, monitoring changes of the ohmic resistance values can be commonly used to track humidity level changes within the membrane, as a lower water content within the membrane is associated with a higher ohmic resistance (membrane dry-out).^30^ In conventional PEMFCs, such an increase in ohmic resistance leads to a linear increase in polarization losses. But for BPIFCs, changes of the ion transport across AEM and PEM or degradation phenomena at the bipolar interface not only impact the linear polarization losses of the ohmic resistance, but additionally influence the water formation reaction at the bipolar junction. Therefore, changes of the ohmic resistance could also be an indicator for the quality of the bipolar junction and possible degradation phenomena which in the end could lead to mass transport restrictions. One particular degradation phenomenon of interest is the delamination of AEM and PEM and thus a change in the PEM/AEM adhesion as schematically depicted in Fig. 9.

**Fig. 10:**
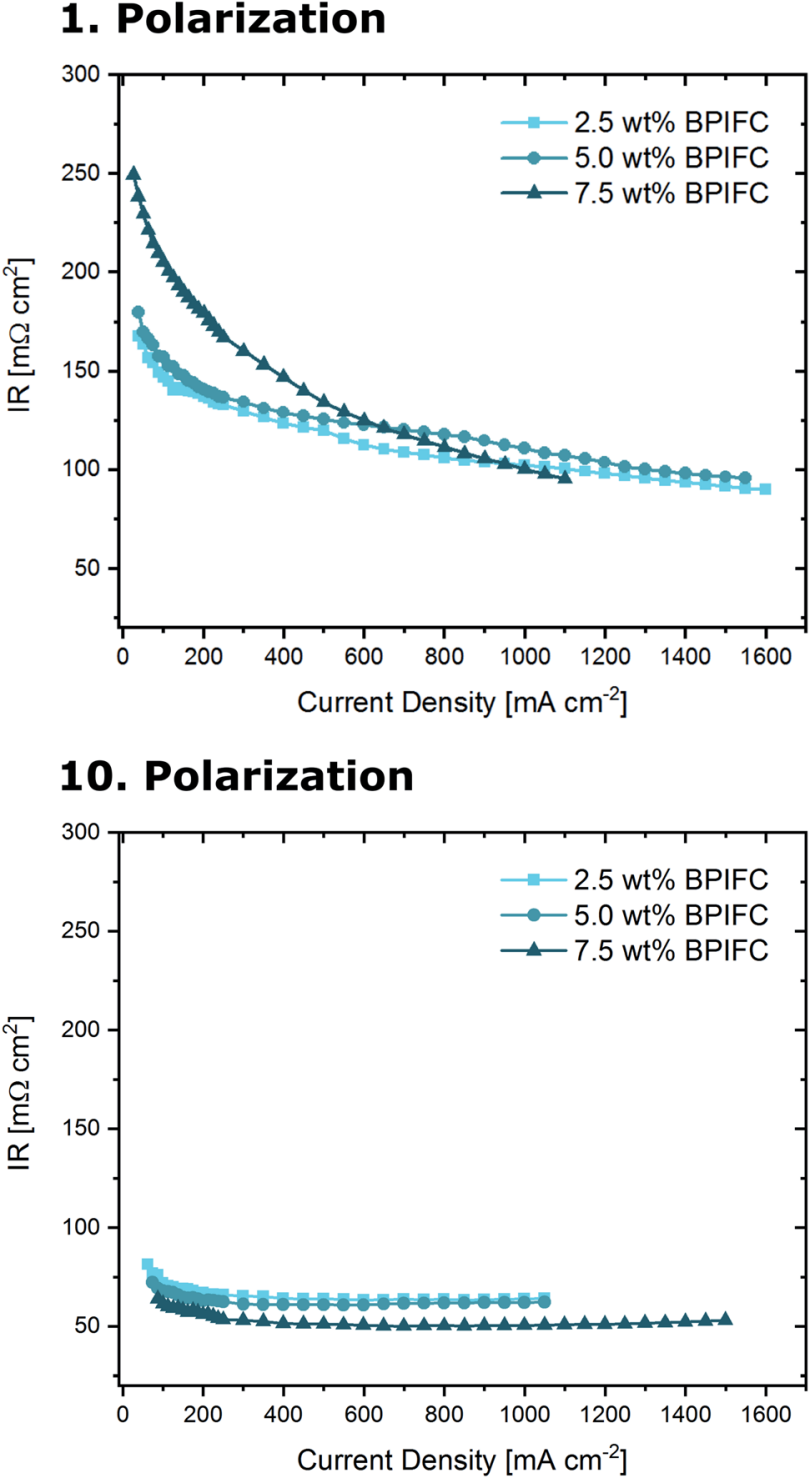
Ohmic cell resistances during the first measured polarization (top) and during the 10 measured polarization (bottom) for the BPIFCs with varying AEM contents. All BPIFCs were tested at 80 °C under O_2_ (0.5 L min^−1^) and H_2_ (0.5 L min^−1^), 100% RH and 200 kPa_gauge_ gas pressure.

One reason for bipolar membrane delamination can be the buildup of hydraulic pressure, when the rate of water removal from the interface through the membranes is lower than the water generation rate. This leads to the accumulation of significant amounts of water in the junction region between the AEM and PEM.^22^ In view of the observed limitations in mass transport, such a degradation phenomenon could be the cause. Theoretically, if a critical current density value is exceeded, water can accumulate in the junction region. This would lead to a hydraulic pressure increase and possibly to the (partial) separation of AEM and PEM. Similar to classical mass transport limiting phenomena, this effect and the resulting overpotential would become more pronounced as the current density is further increased. When such membrane delamination occurs in dependence of the applied current density, the ohmic resistance should increase when approaching the limiting current density values. For that reason, we analyzed *R*_ohmic_ at every recorded point of the oxygen polarization curves of the three different samples (Fig. 10).

For all three cells, the comparison of the initial ohmic resistance values and the values after ten polarizations shows a common trend. The average ohmic cell resistance decreases for all three cells over the course of ten polarizations. This phenomenon is quite common during fuel cell “break-in procedures” and results from the formation of preferential ion conduction paths through the membrane.^31^ As can be seen in Fig. 6, for the 2.5 wt% BPIFC, mass transport limitations began after the first polarization and increased with each subsequent polarization. At the same time, however, a decrease in *R*_ohmic_ was observed after each polarization. For this reason, it is not likely that an ohmic contribution was responsible for the steadily increasing mass transport limiting phenomenon. Therefore, we also exclude a membrane delamination as the cause for the observed mass transport limitation effect.

#### (iii) Porous layer flooding as source for MTL

**Fig. 11:**
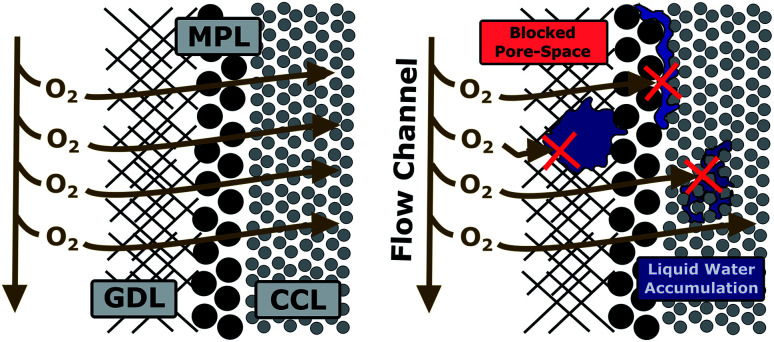
Liquid water accumulation (“flooding”) in the porous layers (GDL, MPL and CCL) as the origin of MTL phenomena.

After excluding bulk electrode gas-phase transport and bipolar interface degradation as the cause of the mass transport limitations observed for the 2.5 wt% BPIFC and the 5.0 wt% BPIFC, the accumulation of liquid water within the porous layers becomes the most likely scenario (Fig. 11).

When now considering porous layer flooding as the cause of the mass transport limitations, it is imperative to take a closer look at the role of the AEM for manipulating the water management within the MEA. More specifically the question occurs: why do the cells with lower AEM content experience more intensive mass transport limitations compared to those with higher AEM content? To approach this question, it is instructive to review the fluidic transport resistance of water through the individual MEA components. Water, which is formed at the bipolar interface, has in principle two ways to be transported out of the system: (i) through the PEM (ii) through the AEM part of the bipolar assembly. Starting with the PEM side it is clear that in all experiments the same acidic half-cell (PEM and anode) was used and remained unchanged throughout the experiments. Therefore, we considered that only an altered state of the AEM and the most likely altered Fe–N/C catalyst layer morphology close to the AEM had an impact on the cell operation behavior as investigated. Consequently, there were two main effects a change in AEM layer thickness and a change in interface pore size that we subsequently discuss.

##### AEM layer thickness – influence of water transport resistance and water uptake

Basically, transport through the polymer-electrolyte membrane reduces the transport rate of the water formed at the bipolar junction because the membrane presents a fluidic transport resistance. On the one hand side this internal fluidic resistance (*R*_fluid_) of the individual membrane layers can be assumed to be directly proportional to its thickness *d*.^32^

1 *R*^AEM^_fluid_ ∝ *d*_AEM_

On the other hand, as the thickness of the AEM layer increases, the absolute amount of water that can be held by the polymeric structure also increases which can be understood like the analogy to an electric capacitance. During the investigation of the self-humidification effect of BPIFCs, Li *et al.* demonstrated that above a certain AEM thickness value, the BPIFC could not be operated without external humidification anymore.^21^ In other words, an increased AEM thickness can reduce the amount of water entering the CCL, where the water is needed for maintaining the ORR. This observed effect can also be applied to the BPIFCs discussed in this paper, but the ultimate consequences are in fact rather different. When comparing the 2.5 wt% BPIFC with the 7.5 wt% BPIFC, the lower absolute amount of AEM material between the CCL and the water recombination sites should result in faster complete saturation of the AEM with water during the break-in procedure. Based on our above discussed equivalence image to electric conduction, the AEM layer takes up water during the first cycles, like a capacitance. Moreover, for further water that is produced at the bipolar junction *R*^AEM^_fluid_ will determine the transport rate of water through the AEM layer.

With *R*_fluid_^2.5 wt%^ < *R*_fluid_^5.0 wt%^ < *R*_fluid_^7.5 wt%^ it becomes obvious that also the water transport rate through the AEM layer of the 2.5wt% BPIFC should be higher than in the other cases.

When combining the effect of a reduced *R*^AEM^_fluid_ and a lower absolute water uptake, in the case of the 2.5 wt% BPIFC, less water can be retained in the membrane itself during the first polarizations and one can assume a higher influx rate of water during the first polarizations into the porous layers. Additionally, the comparatively lower *R*^AEM^_fluid_ of the 2.5 wt% BPIFC results in a higher influx rate of product water into the porous layers also during the following polarizations, which increases the risk for the accumulation of liquid water. Consequently, a possible option would be flooding of the cathode porous layers, caused by too much transport into the cathode side of the cell. For higher wt% the water transport resistance towards the cathode would increase in comparison and more water could also be transported though the acidic anode (PEM and anode CL). Consequently, flooding events might be less frequent or at least delayed in the cathode porous layers.

##### AEM layer thickness – influence on the porous structure of the CCL

Since most of the AEM material infiltrates into the upper part of the CCL during the DMD fabrication process, changed CCL properties in this region are probable, although the bulk electrode oxygen transport properties seem not to be significantly affected as discussed above. Therefore, it may be necessary to consider not only the changes in AEM transport properties, but also to include the water transfer behavior from the AEM layer into the CCL. In the BPIFC system, product water that has successfully passed through the AEM layer can either continue to be transported through the AEI material or enter the open pore space within the CCL. The considerations made for the AEM layer most likely also apply to the AEI material within the CCL. However, additional factors come into play when considering the phase transition from the AEI or AEM material into the pore space of the Fe–N/C layer. As elaborated in previous theoretical work, the CCL properties (dominated by pore size distribution and wettability) might affect the overall water management as the water formation reaction is shifted to the edge of the said layer.^23^ Consequently, the majority of the product water is produced outside of the CCL and the CCL|AEM interface acts as an additional barrier against the water transport. For water to access the pore space of a porous structure, a critical breakthrough pressure must be overcome. The critical break-through pressure is usually expressed according to the Young–Laplace equation:^33,34^2
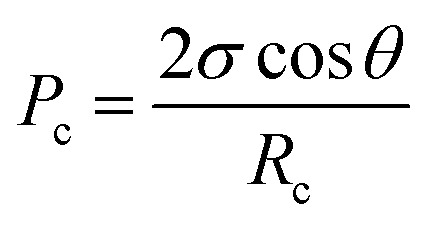
with *P*_c_ = critical break-through pressure, *σ* = surface tension of water, *θ* = contact angle of water within pores and *R*_c_ = capillary radius.

When adding the AEM layer onto the CCL the AEM slurry fills open pore space within the upper region of the Fe–N/C layer and consequently reduces the pore sizes in this part. This reduction of pore size should be dependent on the amount of deposited AEM material. For the 7.5 wt% BPIFC the effect is more pronounced than for the other two cells. According to eqn (2) this pore size reduction results in an increased break-through pressure and hence the transition of product water from the AEI/AEM into the open pore space should be reduced for the 7.5 wt% BPIFC compared to the 2.5 wt% BPIFC at identical conditions. In this case, the water must either diffuse along the AEI for long distances until it reaches the lower part of the catalyst layer, where the local breakthrough pressure is again sufficiently low, or the water pressure builds up in the upper region of the CCL until it can overcome the interfacial resistance of the porous layer. In both cases the transport rate of water into the CCL pore space is reduced.

**Fig. 12:**
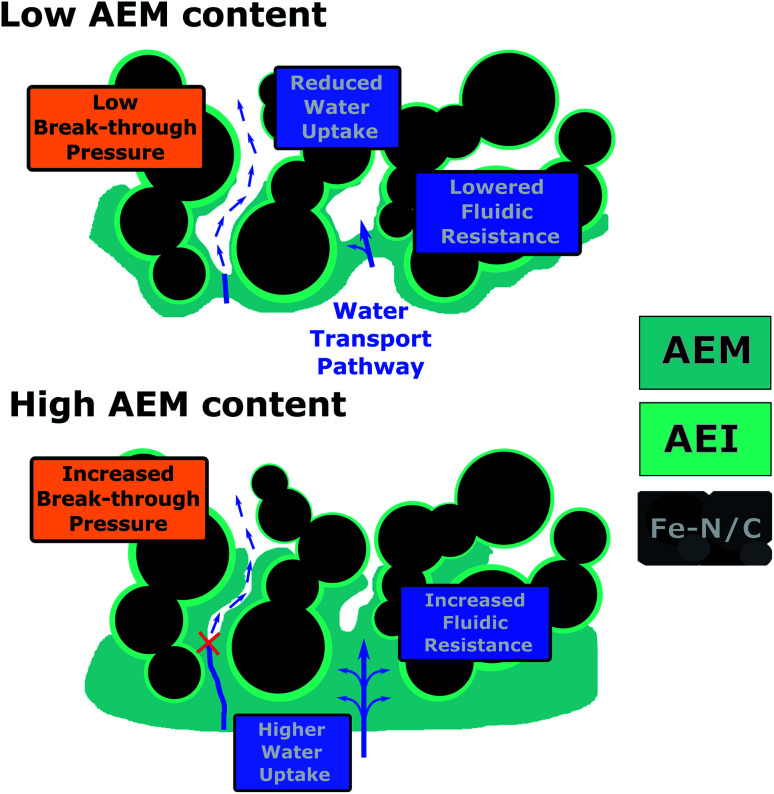
Schematic illustration of the influence of the AEM layer content on the water transport properties through the AEM and into the CCL.

The lower fluidic transport resistance and water uptake of the thinner AEM layer and the lower breakthrough pressure for the CCL of the 2.5 wt% BPIFC compared to the 7.5 wt% BPIFC provide a good theory as to why the low AEM content cells experience stronger and faster onsetting mass transport losses in the case of flooding events (Fig. 12). However, it must be stated, that the proportions of the individual discussed contributions on the overall water content in the porous layers cannot be clarified at this point.

In summary a lower AEM content seems to facilitate the accumulation of liquid water in the porous layers, leading to strong mass transport limitations. At this point, however, it is not clear whether the water accumulation is confined to a specific location within the porous layers. As visible from Fig. 11 the most prominent locations for liquid water accumulation can be the GDL pore space,^35^ the MPL|CCL interface^36^ or within the Fe–N/C layer pore space.^37^

## Conclusion and outlook

This work elaborated in detail how changing the amount of directly deposited AEI on the Fe–N/C-based CCL, that ultimately formed the AEM, gave access to identifying the source of the mass transport limitations (MTL) evident in the polarization data of the lower content AEM BPIFCs (2.5 wt% and 5.0 wt%). Those MTL were a major concern of previous work, as they drastically reduced the power output of the Fe–N/C based BPIFC within several hours. During this work, it could be demonstrated that altering the amount of AEM material does not alter the electrode morphology in a way that gas-phase transport becomes mass transport restricting. A physical delamination of the bipolar interface at high current densities, which would drastically increase the overpotential of the BPIFC, was also excluded. Excessive accumulation of liquid water within the porous layers can be seen as the most likely scenario for the origin of the MTL. The amount of deposited AEM material directly impacts the influx rate of water into the cathode catalyst layer (CCL). The water influx into the CCL was most likely reduced for the high AEM loading cell compared to the low AEM loading BPIFC, as (i) the fluidic resistance of water through the AEM was increased, (ii) the water uptake of the AEM layer was increased and (iii) the porous structure of the CCL is most likely changed in a way that the break-through pressure for water to enter the CCL pore space is increased. For that reason, no MTL were observed for the higher AEM content BPIFC (7.5 wt%) after the break-in procedure. Though the highest AEM loading did not reveal such flooding events during the recording of the polarization data, it cannot be stated generally that a higher AEM loading prevents flooding in total. In fact, there should exist a critical current density value at which the water production rate exceeds the value of water removal. During constant current operation above this critical value, water inevitably accumulates in the porous layers after a certain period of operation time. To prevent such flooding events for Fe–N/C-based BPIFCs, it is necessary to consider the unique role of such thick CCLs for the cell water management. As discussed more thoroughly in previous theoretical work, morphological optimizations of the CCL are necessary to improve its transport properties. Enabling separate pathways for the rapid outflow of product water and for the supply of oxygen and transport of OH^−^ ions through the porous layers seems to be one of the most promising strategies to bypass such strong MTL.

## Experimental

### MEA-preparation

The Pt/C-anode-GDE fabrication was performed according to the routine established and explicitly described in previous work.^16^ For the manufacturing process of the PGM-free high pH cathode a catalyst ink was compounded, comprising of a total 20 wt% solids in 1-propanol. The solid fraction contained 65 wt% Fe–N/C (Pajarito Powder PMF-001602) and 35 wt% AEI (Aemion HNN8-00-X, Ionomr) with an IEC > 2.4. The dissolved AEI was added to the Fe–N/C powder and the resulting ink was mechanically stirred for one hour, placed in an ultra-sonication bath for one hour and after that stirred until usage. An automated film applicator (ZAA 2300, Zehnter) was applied for the deposition of the Fe–N/C ink onto a Freudenberg H23C8 gas diffusion media (4 × 4 cm). The gap-height on the doctor blade controlled the wet-film thickness of the deposited ink-layer. The applicator gap height was set to 200 μm. After the coating process the samples were dried at 40 °C for 2 h on a heating plate. The samples were weighed before and after the catalyst ink deposition and solvent evaporation, to measure the loading of the Fe–N/C GDEs (Sartorius Cubis®, 0.001 mg). The resulting Fe–N/C electrodes had an average catalyst loading of ∼1.2 mg cm^−2^. The AEM layer was directly deposited on the high-pH GDE. Therefore, a 2.5 wt%/5.0 wt%/7.5 wt% dispersion of AEI (Aemion HNN8-00-X, Ionomr) in DMSO (for gas chromatography, Sigma-Aldrich), was applied to the previously prepared GDEs with the automated film applicator and a doctor blade gap height of 100 μm. The samples were dried at 40 °C for four hours and at 40 °C under reduced pressure for two hours. For a complete ion exchange the samples were placed in 1 M KOH for 48 h. The samples were rinsed with H_2_O multiple times in the first step and then placed in H_2_O for 1 h. Before applying the proton exchange membrane, the samples were dried at room temperature. The direct deposition of the proton exchange membrane (on both the anode GDE and cathode GDE), was performed according to the routine established and explicitly described in previous work.^16^

### Fuel cell testing

The fuel cells were assembled and tested according to the routine established and explicitly described in previous work.^16^ For the measurements enabling synthetic air the oxidant flow-rate was set to 0.75 L min^−1^, while keeping the other parameters constant.

## Conflicts of interest

The authors declare no conflict of interest.

## Supplementary Material
